# Ultrasensitive detection of TDP-43 and amyloid-β protein aggregates using micelle-assisted seed amplification assay

**DOI:** 10.1186/s40035-024-00444-7

**Published:** 2024-10-08

**Authors:** Sora Sakamoto, Yuichi Riku, Teiko Komori Nomura, Akio Kimura, Naoki Yamahara, Kazuki Ohuchi, Mari Yoshida, Yasushi Iwasaki, Takayoshi Shimohata, Masatoshi Inden, Ryo Honda

**Affiliations:** 1 Laboratory of Medical Therapeutics and Molecular Therapeutics, Gifu Pharmaceutical University, Gifu, Japan; 2 Department of Neuropathology, Institute for Medical Science of Aging, Aichi Medical University, Nagakute, Japan; 3 Department of Neurology, Nagoya University, Nagoya, Japan; 4 United Graduate School of Drug Discovery and Medical Information Sciences, Gifu University, Gifu, Japan; 5 Department of Neurology, Gifu University Graduate School of Medicine, Gifu, Japan; 6 Center for One Medicine Innovative Translational Research (COMIT), Institute for Advanced Study, Gifu University, Gifu, Japan

## Main text

Ultrasensitive detection of pathological protein aggregates is crucial for early diagnosis and monitoring of neurodegenerative diseases. The seed amplification assay (SAA), a method involving in vitro amplification of pathological aggregates using the native protein monomers as reaction substrates, has proven effective in ultrasensitive detection of prion and α-synuclein (αSyn) protein aggregates in a clinical setting [1]. However, applying this technology to TAR DNA-binding protein 43 (TDP-43) and amyloid-β (Aβ) aggregates remains challenging, because SAA requires a high degree of protein stability of the native monomers that need to function as reaction substrates for an extended period of SAA, typically several days. TDP-43 and Aβ monomers are very unstable proteins that rapidly form liquid droplets, amorphous aggregates, or amyloid fibrils, typically within a few hours after preparation of fresh monomer solutions [2, 3]. Despite efforts by several groups, the limit of detection (LOD) of TDP-43 SAA and Aβ SAA has not exceeded ~ 15 pg (0.4 fmol for TDP-43, 3 fmol for Aβ in monomer equivalent molarity) to date [4–6]. These values, derived using synthetic protein aggregates, inherently have limitations, but they are over 10,000-fold higher than those of prion, αSyn, and tau SAAs with ag to fg sensitivity [1]. Therefore, a general strategy that could enhance LOD by redirecting the unstable aggregation-prone monomers to the seed amplification pathway could further expand the utility of SAA.

This study investigated a novel reagent for SAA with two key properties: (1) the ability to stabilize unstable protein monomers and (2) the capacity to not inhibit seed amplification. The latter property is particularly important in this study, because general protein stabilizers (such as molecular crowders, arginine, etc.), solubilizers (like surfactants, denaturants, etc.), molecular chaperones, and anti-aggregation compounds, while stabilizing protein monomers, may simultaneously inhibit seed amplification. These reagents are designed for protein stabilization and typically act on both native monomers and seed aggregates in a non-specific manner. Interestingly, there has been limited research into SAA-specific reagents, with the exception of recent studies on Hofmeister salts [7] and the development of small compounds for αSyn SAA [8].

**Fig. 1:**
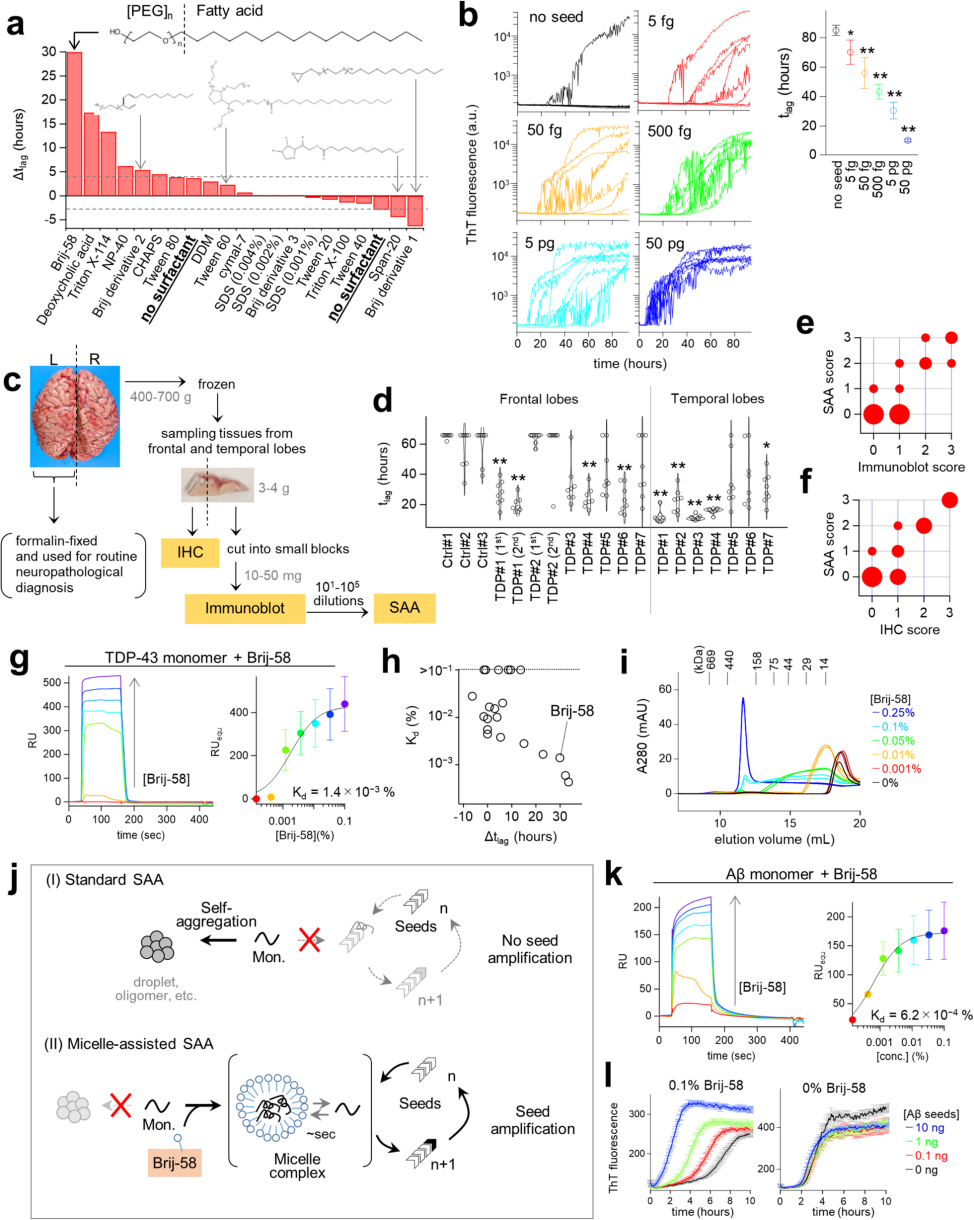
**a** Screening result of surfactants at the concentration of 0.1% unless otherwise noted. **b** Performance of the enhanced TDP-43 SAA. The left panel shows the time courses of ThT fluorescence at different seed concentrations. The right panel presents* t*_lag_ values derived from the left panel (mean ± SE, *P < 0.05, **P < 0.005, unpaired two-tailed *t*-test compared to no seed). **c** Outline of the experiment using FTLD-TDP patients’ brains. Small sections from the frontal and temporal lobes of frozen brains in the right hemisphere were sampled and subjected to SAA, immunoblotting, and IHC. **d** The* t*_lag_ values of the patient-derived brain homogenates obtained at a 1:250 dilution ratio (*P < 0.05, **P < 0.005, Dunnett’s test compared to control brain homogenate). Frontal lobes from the TDP#1 and #2 patients were evaluated in two different extracts. **e**, **f** Bubble plots showing the correlation of SAA with immunoblot and IHC scores. The size of the bubbles is proportional to their frequency. **g** Left panel: SPR sensorgrams of Brij-58 binding to TDP-43 monomers. Right panel: a dose–response curve derived from the left panel. **h** Correlation between ∆*t*_lag_ and *K*_d_ values of 21 surfactants. **i** SEC profiles of 20 µM TDP-43 monomers mixed with Brij-58 prior to analysis. **j** Schematic of how Brij-58 redirects aggregation-prone protein monomers to the seed amplification pathway. **k** SPR sensorgrams of Brij-58 binding to Aβ monomers. **l** Time courses of ThT fluorescence of Aβ SAA (with 1.5 M urea) at different seed concentrations, both without and with 0.1% Brij-58

Here, we focused on a series of surfactants based on our preliminary screening results and on the previous reports showing that sodium dodecyl sulfate (SDS) enhances the LOD of prion SAA [9]. We evaluated a variety of surfactants using a standard TDP-43 SAA that incorporated a recombinant TDP-43 C-terminal domain fragment (aa. 267 − 414), a low-salt buffer, and thioflavin-T (ThT) (Fig. S1a). We measured sensitivity by observing the difference in the lag time of protein aggregation with or without synthetic TDP-43 seed fibrils (∆*t*_lag_) (Fig. S1b, c). Interestingly, Brij-58, a linear polyethylene glycol (PEG) ester of palmitic acid, dramatically increased the ∆*t*_lag_ value (Fig. 1a). Brij-58 gave a bell-shaped dose–response curve with an optimal concentration range of 0.02%–0.2% (Fig. S1d), which was much higher than the critical micelle concentration (CMC) (0.001%) (Table S1), indicating that the micelle formation plays a role in its mechanism-of-action (MoA). In contrast, SDS showed no activity toward TDP-43 SAA, both at high (0.1%) and at the previously reported concentrations below CMC (0.001%–0.004%) [9]. Tween-60 and other Brij-58 analogs showed no activity, indicating that the linear structure composed of PEG and fatty acid is crucial for the observed activity. The structure–activity relationship analysis revealed that the conjugation of 20–23 PEG repeats with either palmitic acid (C16:0) or stearic acid (C18:0) yielded the best activity (Fig. S1e).

In efforts to further improve the sensitivity, we discovered that addition of 0.5–1.0 M urea, but not guanidine hydrochloride (GuHCl), further increased the ∆*t*_lag_ value (Fig. S1f). Overall, the enhanced TDP-43 SAA was able to detect seed fibrils as low as 5 fg seeds (Fig. 1b). Furthermore, the *t*_lag_ value showed an excellent correlation with seed concentration, indicating a dynamic range from 5 fg to 50 pg. Notably, the TDP-43 SAA did not detect Aβ or αSyn seed fibrils at 1 ng (Fig. S2). Therefore, TDP-43 SAA is a sensitive and specific assay for detecting TDP-43 seed fibrils.

To assess applicability of our method to patient-derived TDP-43 aggregates, we examined autopsied brain tissues from patients diagnosed with frontotemporal lobar degeneration (FTLD) (FTLD-TDP type A *n* = 3, type B *n* = 4) and from neurologically healthy control patients (*n* = 3) (Fig. 1c, Tables S2 and S3). The TDP-43 SAA with Brij-58 detected varying degrees of TDP-43 seeding activity depending on the patient and the sampling site (Fig. 1d and Fig. S3), while without Brij-58, it did not (Fig. S4). Overall, seeding activity was detected in six out of seven (85.7%) FTLD-TDP patients in either frontal or temporal lobe-derived homogenate at a dilution of 1:250. A single case (TDP#1) exhibited prominent TDP-43 seeding activity, detectable at dilutions as low as 10^−5^ (Fig. S5a and S6). No seeding activity was detected in the three control samples (Fig. 1d) or in four additional control samples from a second cohort (Fig. S7). Notably, the measured seeding activity correlated well with the results of standard immunoassays probing phosphorylated TDP-43, namely immunoblots and immunohistochemistry (IHC) (Fig. 1e, f, Fig. S5b, and Fig. S8). Samples from patient TDP#5 were negative or minimally positive in both SAA and immunoassays, suggesting that the sampled sections contain very few seeding-active TDP-43. Thus, our SAA protocol using Brij-58 is effective for detecting patient-derived TDP-43 aggregates.

Brij-58 dramatically accelerated in vitro amplification of TDP-43 seed fibrils (Fig. S9 and S10). To investigate its unique MoA, we used surface plasmon resonance (SPR) to study the interactions between the TDP-43 monomer and the 21 surfactants. Brij-58 and most surfactants showed a very rapid, reversible, and nearly 1:1 binding kinetics to the TDP-43 monomer (Fig. 1g and S11). Interestingly, the dissociation constants of binding (*K*_d_) values correlated well with the ∆*t*_lag_ values in SAA (Fig. 1h). Therefore, the strength of the molecular interaction between TDP-43 monomers and surfactants is a major determinant of activity.

We further investigated the interactions between Brij-58, TDP-43 monomers, and seed fibrils using structural biology methods, namely liquid droplet assay (Fig. S12), far-UV circular dichroism (Fig. S13), size exclusion chromatography (SEC) (Fig. 1i and S14), transmission electron micrography (Fig. S15a), limited proteolysis (Fig. S15b), and GuHCl-unfolding assay (Fig. S15c). Overall, we found that (1) Brij-58 and TDP-43 monomers formed a rapidly reversible 200 kDa micelle complex that inhibits the self-aggregation of TDP-43 monomers, and (2) Brij-58 did not disrupt the stability or structure of TDP-43 seed fibrils, thereby specifically acting on TDP-43 monomers. Therefore, Brij-58 redirects the aggregation-prone TDP-43 monomers to seed amplification pathway, making them suitable as SAA substrates (Fig. 1j).

We hypothesized that Brij-58 could also bind to Aβ monomers due to two reasons: (1) the interaction between surfactants and proteins is generally driven by non-specific hydrophobic interactions, and (2) Aβ is a highly hydrophobic peptide prone to aggregation. Indeed, SPR analysis showed that Brij-58 bound tightly to Aβ monomers with a *K*_d_ value lower than that of TDP-43 monomers (Fig. 1k). Furthermore, an enhanced Aβ SAA, which employed a combination of 0.1% Brij-58 and 0.5 − 1.5 M urea, could detect seed fibrils at levels as low as 100 pg (Fig. 1l). Without Brij-58, the LOD did not exceed 10 ng, indicating that Brij-58 improved the sensitivity of Aβ SAA by over 100-fold (Fig. S16). In contrast, Brij-58 did not bind to hydrophilic αSyn, or improve the LOD of αSyn SAA (Fig. S17).

In summary, Brij-58 can be utilized for multiple SAAs that are challenged by the hydrophobic and aggregation-prone nature of the substrate monomers, including TDP-43, Aβ and possibly other unstable proteins, such as FUS, EWSR1, and TAF15. The interaction between Brij-58 and protein monomers, followed by the formation of a rapidly reversible 200 kDa micelle complex, is crucial for its MoA. Due to the monomer-dependent MoA, its effect largely depends on the properties of the substrate monomer, rather than the origin of the seeds whether synthetic or patient-derived. Notably, the LOD of the enhanced TDP-43 SAA is as low as 5 fg, which is comparable to prion and αSyn SAAs currently used in clinical settings. We anticipate further enhancements of SAA down to ag or few-molecule levels through integration with immunoprecipitation, proteolytic cleavage of protein aggregates, and engineering of substrate monomer. Efforts are also underway to translate our TDP-43 SAA into clinical practice by detecting TDP-43 aggregates in clinically accessible specimens, such as cerebrospinal fluid and blood.

## Supplementary Information

Additional file 1. **Table S1**. Basic properties and activities of surfactants.

Additional file 2. **Table S2**. TDP-43 detection from frozen brain using IHC, immunoblot, and SAA.

Additional file 3. **Table S3**. Patients' demography.

Additional file 4. **Material and methods**. **Figure S1**. Development of an ultrasensitive TDP-43 SAA. **Figure S2**. Seed-specificity of the enhanced TDP-43 SAA. **Figure S3.** Original time courses of ThT fluorescence in the presence of patient-derived brain homogenates (1:250 dilution). **Figure S4**. Effects of Brij-58 in the detection of TDP-43 aggregates from patient-derived brain tissues. **Figure S5**. Detection of TDP-43 aggregates in patient-derived brain tissues using SAA and immunoblot. **Figure S6**. Original time courses of ThT fluorescence in the presence of patient-derived brain homogenates (1:1250–156250 dilutions). **Figure S7**. A result of TDP-43 SAA for four neurologically healthy controls in a second cohort. **Figure S8**. IHC images of brain tissues probing phosphorylated TDP-43 at Ser409/410. **Figure S9**. Brij-58 dramatically accelerates amplification of TDP-43 seed amplification. **Figure S10**. Original time courses of ThT fluorescence in the presence of 16 ng seeds and 1 M urea. **Figure S11**. SPR sensorgrams and dose-response curves of surfactant. **Figure S12**. Liquid droplet assay. **Figure S13**. Far-UV circular dichroism (CD) analysis of the Brij-58/TDP-43 complex. **Figure S14**. Size exclusion chromatography (SEC) analysis of the Brij-58/TDP-43 complex. **Figure S15**. Brij-58 does not significantly affect the structure or stability of seed fibrils. **Figure S16**. Brij-58 improves the detection limit of Aβ SAA. **Figure S17**. Brij-58 did not bind to αSyn, nor improves the detection limit of αSyn SAA.

## Data Availability

Not applicable.

## Abbreviations

SAA: Seed amplification assay
LOD: Limit of detection
CMC: Critical micelle concentration
TDP-43: TAR DNA-binding protein 43
MoA: Mechanism-of-action
ThT: Thioflavin-T
SEC: Size exclusion chromatography
FTLD: Frontotemporal lobar degeneration
GuHCl: Guanidine hydrochloride
PEG: Polyethylene glycol
SPR: Surface plasmon resonance
SDS: Sodium dodecyl sulfate
