# Supplementary material for: Ultrasensitive detection of TDP-43 and amyloid-β protein aggregates using micelle-assisted seed amplification assay
Source: Transl Neurodegener. 2024 Oct 8;13:51. doi: 10.1186/s40035-024-00444-7 (PMC11460235; doi:10.1186/s40035-024-00444-7)
Supplement: Supplementary file 4 — Additional file 4. Material and methods. Figure S1. Development of an ultrasensitive TDP-43 SAA. Figure S2. Seed-specificity of the enhanced TDP-43 SAA. Figure S3. Original time courses of ThT fluorescence in the presence of patient-derived brain homogenates (1:250 dilution). Figure S4. Effects of Brij-58 in the detection of TDP-43 aggregates from patient-derived brain tissues. Figure S5. Detection of TDP-43 aggregates in patient-derived brain tissues using SAA and immunoblot. Figure S6. Original time courses of ThT fluorescence in the presence of patient-derived brain homogenates (1:1250–156250 dilutions). Figure S7. A result of TDP-43 SAA for four neurologically healthy controls in a second cohort. Figure S8. IHC images of brain tissues probing phosphorylated TDP-43 at Ser409/410. Figure S9. Brij-58 dramatically accelerates amplification of TDP-43 seed amplification. Figure S10. Original time courses of ThT fluorescence in the presence of 16 ng seeds and 1 M urea. Figure S11. SPR sensorgrams and dose-response curves of surfactant. Figure S12. Liquid droplet assay. Figure S13. Far-UV circular dichroism (CD) analysis of the Brij-58/TDP-43 complex. Figure S14. Size exclusion chromatography (SEC) analysis of the Brij-58/TDP-43 complex. Figure S15. Brij-58 does not significantly affect the structure or stability of seed fibrils. Figure S16. Brij-58 improves the detection limit of Aβ SAA. Figure S17. Brij-58 did not bind to αSyn, nor improves the detection limit of αSyn SAA. [file 40035_2024_444_MOESM4_ESM.docx]

**Additional file 1**

**Ultrasensitive detection of TDP-43 and Amyloid-β protein aggregates using micelle-assisted seed amplification assay**

Sora Sakamoto^1^, Yuichi Riku^2,3^, Teiko Komori Nomura^4^, Akio Kimura^5^, Naoki Yamahara^5^, Kazuki Ohuchi^1^, Mari Yoshida^2^, Yasushi Iwasaki^2^, Takayoshi Shimohata^5,6^, Masatoshi Inden^1^, and Ryo Honda^4,6^*

^1^Laboratory of Medical Therapeutics and Molecular Therapeutics, Gifu Pharmaceutical University; ^2^Department of Neuropathology, Institute for Medical Science of Aging, Aichi Medical University; ^3^Department of Neurology, Nagoya University; ^4^United Graduate School of Drug Discovery and Medical Information Sciences, Gifu University; ^5^Department of Neurology, Gifu University Graduate School of Medicine; ^6^Center for One Medicine Innovative Translational Research (COMIT), Gifu University.

# *To whom corresponding should be addressed: [ryohonda.rh@gmail.com](mailto:ryohonda.rh@gmail.com)**Material and methods**

**Surfactants**

Surfactants were purchased from various vendors as listed in Table S1. Prior to use, the surfactants were dissolved with distilled water at the concentration of 1–10% w/v depending on their solubility. Water-insoluble surfactants (Brij S10 and Brij C10) were dissolved with ethanol at the concentration of 10% w/v.

**Measurement of critical micelle concentration (CMC)**

The critical micelle concentrations (CMC) of surfactants were determined using Nile Red (NR) dye as described previously [1]. Briefly, serial dilutions of surfactants (final concentrations of 0.0014, 0.0041, 0.012, 0.037, 0.11, 0.33, 1%) that were dispersed with distilled water were added with NR at the final concentration of 1 µM. After 30-minute incubation at room temperature, fluorescence intensity at 640 nm (exaction wavelength, 580 nm) was recorded on SpectraMax iD5 Multi-Mode Microplate Readers (Molecular Devices). Fluorescence intensity was plotted as a function of the surfactant concentration and fitted to a sigmoid function using Igor Pro 9.02. The second derivative was used to determine the surfactant concentration at the lower inflection point, which represents the CMC. The experiments were repeated four times to estimate the lower and upper limits of CMC (average ± 2 S.E.).

**Preparation of recombinant TDP-43 proteins**

The pRSET A Bacterial Expression Vector (Thermo Fisher Scientific #V35120) was inserted with a DNA fragment encoding human TDP-43 (aa. 267–414) at NheI and BamHI sites. For preparation of a fluorescently labeled protein, the Cys 275 variant was generated by site-directed mutagenesis. The plasmids encoding the His-tagged TDP-43 proteins were transformed into ECOS Competent *E. coli* BL21 using the manufacturer's protocol (NIPPON GENE #314-06533). The next day, a single colony was inoculated into 5 mL of LB medium containing 100 µg/mL ampicillin and pre-cultured at 37°C with 180–240 rpm shaking until mid-log phase. The pre-cultured E. coli was inoculated into 1.25 L of LB medium containing 50 µg/mL ampicillin and shaked at 37°C with 180–240 rpm until mid-log phase. Protein expression was induced by 1 mM IPTG followed by 4 h shaking at 37°C. The *E. coli* was harvested by centrifugation at 7,000 g for 12 min, resuspended with 20 mM Tris-HCl (pH 8) buffer, and sonicated on ice using VP-300N ultrasonic processor (TAITEC, #0075955-001). The disrupted E. coli was centrifuged at 30,000 g for 25 min, and the supernatant fraction was discarded. The pellet fraction was solubilized in 6 M GdHCl/50 mM Tri-HCl buffer (pH 8) with 100 rpm shaking at room temperature for 3–24 h. The solution was subjected to centrifugation at 30,000 g for 60 min, and the supernatant was collected for the subsequent purification procedure.

The supernatant containing solubilized proteins was initially purified using an immobilized metal affinity chromatography (IMAC) that uses 7.5 M urea to prevent protein aggregation during purification. Briefly, 5 mL of Ni Sepharose 6 Fast Flow resin (Cytiva) was loaded into a spin column, washed with 10 mL deionized water, and charged with 10 mL 0.1 M NiSO_4_. The column was washed again with 10 mL deionized water and equilibrated with 10 mL binding buffer [100 mM sodium phosphate, 10 mM Tris-HCl (pH 7.8), 20 mM imidazole, and 7.5 M urea]. Then, the solubilized protein was applied to the column and extensively washed with 10 mL of binding buffer until the absorbance at 280 nm of the flow-through was less than the detection limit. The protein was eluted by applying 10 mL elution buffer [100 mM sodium phosphate, 10 mM Tris-HCl (pH 7.8), 500 mM imidazole, and 7.5 M urea] to the resin.

The IMAC-purified protein was further purified by reverse-phase chromatography using COSMOSIL Protein-R column (Nacalai Tesque #06532-31) connected to AKTApurifier (Cytiva). Proteins were eluted with a linear gradient of 20–55% acetonitrile in 0.1% trifluoroacetic acid. A fraction enriched with TDP-43 was collected and lyophilized to remove the solvent. The lyophilized protein was dissolved with 6 M GuHCl at a concentration of 500 µM to remove any pre-existing aggregates. The protein concentration was determined using UV absorbance at 280 nm based on the predicted extinction coefficients (18,350 cm^−1^M^−1^). The stock solution was passed thorough 0.22 µm filter, dispensed into aliquots, and stored at −80ºC until use.

**Preparation of recombinant amyloid β**

The pET-28c(+) vector (Merck #69866) was inserted with a DNA fragment encoding human amyloid β (aa. 1–42) at NcoI and SalI sites. The plasmid encoding amyloid β protein with starting methionine was transformed into *E. coli* BL21 and the protein expression was induced as described above. The disrupted *E. coli* was centrifuged at 30,000 g for 25 min, and the supernatant fraction was discarded. The pellet fraction was washed three times with a Triton X-100 buffer (20 mM Tris-HCl, pH 8, and 4% Triton X-100), washed twice with 20 mM Tri-HCl buffer (pH 8), and solubilized in 7.5 M Urea/20 mM Tri-HCl buffer (pH 8) with 100 rpm shaking at room temperature for 3–24 h. The solution was subjected to centrifugation at 30,000 g for 60 min, and the supernatant was collected for the subsequent purification procedure.

The solubilized protein was purified using a cation-exchange affinity chromatography with a standard procedure. Briefly, the protein was applied to Q Sepharose Fast Flow resin (Cytiva) loaded on a column, washed three times with 100 mM NaCl buffer (7.5 M Urea, 20 mM Tri-HCl buffer, pH 8), and eluted with 200 mM NaCl buffer (7.5 M Urea, 20 mM Tri-HCl buffer, pH 8). The purified protein was concentrated by ultrafiltration (Millipore, 3,000 MWCO) and stored at −80 ºC. Just prior to use, the protein was applied to Superdex 75 10/300 GL column (Cytiva) equilibrated with 10 mM sodium phosphate (pH 7.4) buffer. A fraction containing protein monomers was collected and immediately used for SAA.

**Preparation of recombinant α-synuclein proteins**

The pET-23a(+) vector (Merck #69745) was inserted with a DNA fragment encoding human α-synuclein (aa. 1–140) at NdeI and XhoI sites. The codon 136 was mutated from TAC to TAT to prevent incorporation of a Cys residue instead of a Tyr [2]. The expression and purification of α-synuclein proteins was performed as described previously [3]. The lyophilized protein was dissolved with 6 M GuHCl at a concentration of 2 mM to remove any pre-existing aggregates. Protein concentration was determined using UV absorbance at 280 nm based on a predicted extinction coefficient (5,140 cm^−1^M^−1^). The stock solution was passed thorough 0.22 µm filter, dispensed into aliquots, and stored at −80ºC until use.

**Seed amplification assay (SAA)**

Just prior to the assay, the above-described protein stock solutions were diluted with a reaction buffer [20 mM HEPES-NaOH (pH 7.2), 1 mM EDTA, 0.01% NaN_3_, and 25 µM Thioflavin-T] supplemented with denaturant, salts, surfactants, and PEG as necessary. The final protein concentrations were 5 µM for TDP-43, 50 µM for α-synuclein, and 3 µM for amyloid-β. Then, the reaction mixture containing protein monomers was mixed with test solutions containing seeds fibrils or patient-derived brain homogenates on a 96-well black microplates (Corning #3650, #3993, or PerkinElmer-OptiPlate #6055260). After mixing, the plate was tightly sealed with transparent film (WATSON, Cat. No. 547-KTS-HCP), and the time courses of ThT fluorescence were recorded by top-reading on the TECAN Infinite 200 F Nano+ with an excitation filter of 435 nm and an emission filter of 485 nm. Shaking condition and temperature were as follows; for TDP-43 and α-synuclein SAAs, continuous orbital shaking at 176 rpm and 42ºC; for amyloid-β SAA, repeated cycle of incubation and shaking (5-min incubation followed by 10-sec orbital shaking at 176 rpm) at 37ºC.

Seed elongation assay shown in Figures S7 and S8 was performed using a similar procedure. Briefly, 10 µl of solution containing 160 µg/ml TDP-43 seed fibrils (10 µM in monomer equivalent unit) was mixed with 90 µl of the reaction mixture containing 5 µM TDP-43 monomers. The time courses of ThT fluorescence were recorded at 37ºC under a quiescent condition without shaking. Initial rates were determined by fitting the initial region of each growth curve to a double-exponential function and taking the first derivatives of the function at time zero.

**Preparation of seed fibrils**

The TDP-43 seed fibrils were prepared in the SAA reaction buffer [20 mM HEPES-NaOH (pH 7.2), 1 mM EDTA, 0.01% NaN_3_] at a protein concentration of 10 µM (160 µg/ml), supplemented with Brij-58 as necessary, and rotated at 37ºC for 1–5 days. The α-synuclein seed fibrils were prepared in a phosphate-buffered saline [20 mM NaPi (pH 7.4), 150 mM NaCl, and 0.01% NaN_3_] at a protein concentration of 300 µM (4.2 mg/ml), and rotated at 37ºC for 5–7 days. The amyloid-β seed fibrils were prepared in the same SAA reaction buffer at a protein concentration of 20 µM (80 µg/ml) by repeated cycles of repeated cycle of incubation and shaking (5-min incubation followed by 10-sec orbital shaking at 176 rpm) at 37ºC in the 96-well black microplate. The prepared seed fibrils were dispensed into aliquots, and stored at −80ºC until use. Just prior to use, the seed solutions were thawed on ice and diluted with distilled water to various concentrations. The α-synuclein seed fibrils were sonicated for 30 s at 30% power on ice using VCX-130 (EYELA), while the TDP-43 and amyloid-β seed fibrils were used without sonication.

**Transmission Electron microscopy (TEM)**

Following the previously published protocol [4], a solution containing seed fibrils was 10-fold diluted with deionized water and immediately loaded onto a carbon-coated grid (EM Japan, Cat. No. U1013). The grid was negatively stained with 2% phosphotungstate (pH 7) and examined using a transmission electron microscope (JEOL, JEM-2100 F).

**Preparation and analysis of patient-derived brain homogenates**

We included seven patients with sporadic ALS/FTLD-TDP and seven neurologically healthy controls. The cases were autopsied at the Institute of Medical Science for Aging, Aichi Medical University, Japan. Research-purpose archiving of autopsied tissues was approved by the ethical committee of the university. For all patients, the left hemisphere, brainstem, cerebellum, and spinal cord were fixed in 10% formaldehyde for at least two weeks and then, embedded in the paraffin after trimming; the blocks were sectioned in 5 μm-thickness to make neuropathological diagnoses. The right hemisphere was frozen; tissues of 3–4g were sampled from the frontal lobe (premotor cortex) and temporal lobe (middle temporal gyrus), which were subjected to SAA and immunoblotting. We also fixed a part of the sampled frozen tissue in 10% formaldehyde for immunohistochemistry. It allowed us to directly assess the presence of TDP-43 aggregates within the samples that were subjected to SAA and immunoblotting.

For SAA and immunoblot analysis, sarkosyl-insoluble fractions were extracted from the frozen tissues, following the previously published protocol [4]. Briefly, 10–50 mg of the frozen tissues were crushed using an AUTOMILL machine (Tokken Inc. #TK-AM7), added with 50 volumes (v/w) of an extraction buffer [10 mM Tris-HCl, pH 7.5, 800 mM NaCl, 10% sucrose, 1 mM EGTA] supplemented with 2% sarkosyl, and rotated for 60 min at 37 °C. Following 15 min of centrifugation at 20,600 g at 25°C, the supernatants were centrifuged at 217,000 g for 25 min at 25°C. Then, the pellets were resuspended in 100 μl of the extraction buffer containing 1% sarkosyl by sonication for 15 min on an ultrasonic cleaner (AIWA, #AU-16C) and centrifuged at 10,000 g for 5 min at 25 °C. The supernatants were collected and centrifuged at 217,000 g for 25 min, and the resulting pellets were resuspended in an extraction buffer containing 1% sarkosyl as described above. For immunoblotting, 8 μl of the resuspension was mixed with 2 μl of 5×sample buffer [0.25 M Tris-HCl (pH 6.8), 10% SDS, 0.5% Bromophenol Blue, 0.5 M DTT, and 50% glycerol], boiled for 5 min, and subjected to immunoblot analysis, as previously described [5]. The membrane was probed with a primary antibody (Anti-TDP-43, phospho Ser409/410, clone 11-9, COSMO BIO #TIP-PTD-M01A) and reacted with a secondary antibody (Anti-mouse IgG, HRP-linked Antibody, CST #7076). The band intensities were semi-quantified by the Image J software. For SAA, the remaining resuspension was further centrifuged at 10,000g for 5 min at 25 °C. The supernatants were collected and centrifuged at 217,000 g for 25 min, and the resulting pellets were resuspended in an extraction buffer containing 1% sarkosyl as described above. The resuspension was stored at −80ºC until use, and diluted with distilled water just prior to TDP-43 SAA. The SAA score was defined as follows: grade −, negative at dilution ratio of 1:250; grade +, positive at dilution ratio of 1:250 but negative at dilution ratio of 1:1250; grade ++, positive at dilution ratio of 1:1250 but negative at dilution ratio of 1:6250; grade +++, positive at dilution ratio of 1:6250.

For Immunohistochemistry, we used the primary antibodies as follows: anti-alpha-synuclein (rabbit polyclonal, 1:2000, Sigma-Aldrich, St. Louis, MO), anti-beta-amyloid (12B2, mouse monoclonal, 1:500, Immuno-Biological Laboratories, Minneapolis, MN), anti-hyperphosphorylated tau (AT8, mouse monoclonal, 1:3000, Thermo Fisher Scientific, Waltham, MA), anti-panTDP-43 antibody (rabbit polyclonal, 1:2500, Proteintech, Chicago, IL), anti-phosphorylated TDP-43 (p-TDP-43 s409/410, rabbit polyclonal, 1:3000, Cosmobio, Tokyo, Japan). Secondary immunolabeling was performed using biotinylated immunoglobulins by the standard avidin-biotin method. We semi-quantified density of TDP-43 aggregates from randomly chosen five visual fields under ×20 objective as follows; grade 0, no aggregates in all five visual fields; grade +, neuronal cytoplasmic inclusions were found in one or two out of five visual fields; grade ++, neuronal cytoplasmic inclusions were found in three or four out of five visual fields; and grade +++, neuronal cytoplasmic inclusions were found in all five visual fields. Anti-hyperphosphorylated tau and anti-β amyloid immunohistochemistry revealed aging-related pathology in some patients, which were below diagnostic cut-off of Alzheimer disease (Braak tau stages ranged from 0 to III, and Thal’s amyloid phase ranged from 0 to 4).

**Surface plasmon resonance**

Direct interactions between protein monomers and surfactants were analyzed using a BIACORE T200 (GE Healthcare) as previously described [3]. Briefly, the recombinant TDP-43 CD, amyloid-β, α-synuclein were immobilized into a CM5 sensorchip using an amine-coupling kit at a density of approximately 3,000, 3000, and 1000 response units (RU), respectively. An adjacent flow cell was blocked with ethanolamine and utilized as a reference surface. A standard HEPES-buffered saline without surfactants was used as a running buffer. The surface was injected over with surfactants at various concentrations with a flow rate of 30 μL/min at 20°C. A plot of RU values at 120 secs versus surfactants concentration was fitted to a sigmoidal function to derive dissociation constant (K_d_):

$$\left[ RU \right]=\frac{{RU}_{max}}{1+K_{d}/[surfactant concentraiton]}$$

**Analysis of TDP-43 droplets**

A fluorescently labeled TDP-43 CD was prepared by attaching Fluorescein-5-maleimide (TCI Chemicals, #F0810) to the protein variant harboring Cys 275, following previous published protocols [5,6]. Briefly, after the IMAC purification, 25 µM TDP-43 (C275) was treated with 1 mM TCEP in the IMAC elution buffer [100 mM sodium phosphate, 10 mM Tris-HCl (pH 7.8), 500 mM imidazole, and 7.5 M urea] for 30 min at RT. Subsequently, 125 µM Fluorescein-5-maleimide was added and incubated for 2 hours at RT in a dark room. The reaction mixture was washed 5 times using Amicon Ultra 10K centrifugal filter devices with a wash buffer [100 mM acetate (pH 4.0) and 7.5 M urea] at 4°C. The fluorescently label TDP-43 CD was purified by PRC and lyophilized using the procedure described in the above section.

Just prior to use, the lyophilized protein with or without fluorescein was dissolved with distilled water. For the determination of the concentrations of fluorescently labeled proteins, the solution was 10-fold diluted with an acidic buffer (50 mM acetate, pH 4.0), and the absorbance at 280 and 494 nm was measured. The concentrations of protein and fluorescein were determined using the formula:

$$\left[ protein \right]=\frac{A_{280}-A_{494}\times CF}{\varepsilon_{protein}}$$

$$\left[ fluorescein \right]=\frac{A_{494}}{\varepsilon_{fluorescein}}$$

We used *ε_protein_* = 18,350 cm^−1^M^−1^, *ε_fluorescein_* = 23,000 cm^−1^M^−1^, and CF = 0.77 in the formula. The latter two values at pH 4.0 were determined in-house by comparing the absorbance of a standard fluorescein solutions between pH 4.0 and pH 7.4 with *ε_fluorescein_* = 68,000 cm^−1^M^−1^ at pH 7.4. Based on the formula, typical labeling ratio ([fluorescein]/[protein]) was calculated to be 1.0~1.3.

For the turbidity measurement, solutions containing non-labeled TDP-43 monomers at various protein concentrations were mixed with buffers [20 mM HEPES-NaOH (pH 7.2), 1 mM EDTA, 0.01% NaN_3_, and varying concentrations of NaCl] and transferred to a clear-bottom half area 96-well plate (Grainer #675100). After 1-hour incubation at 37°C, the absorbance at 600 nm was measured by the TECAN Infinite 200 F Nano+. For the fluorescence microscopy measurement, a solution containing fluorescein-TDP-43 was mixed with a buffer [20 mM HEPES-NaOH (pH 7.2), 1 mM EDTA, 0.01% NaN_3_, and 300 mM NaCl] at the final protein concentration of 20 µM. After 5 min incubation at RT, the mixture was dropped on a coverslip and examined on a fluorescence microscopy (KEYENCE, BZ-9000) with a GFP filter (excitation 470/40 nm and emission 525/50 nm).

**Circular dichroism (CD) measurements**

The secondary structure of 5 µM TDP-43 CD was examined at 25 °C using a Chirascan-plus CD spectrometer (Applied Photophysics) with a 1 mm path length quartz cuvette (Hellma, 100-QS). Just prior to the measurement, the lyophilized TDP-43 protein was dissolved with distilled water, mixed with various concentrations of Brij-58 in the presence of 10 mM sodium phosphate (pH 7.2), and incubated for 10 min at 25°C.

S**ize exclusion chromatography (SEC) analysis**

The SEC experiments were performed using a Superdex increase 200 10/300 column (Cytiva) connected to the AKTApurifier. A HBS buffer (10 mM HEPES, pH 7.4, 150 mM NaCl) was used as a running buffer at the flow rate 1.0 mL/min at RT. Just prior to the measurement, the stock solution (containing 500 µM TDP-43 CD and 6 M GuHCl) was 25-fold diluted with the HBS buffer with or without Brij-58, incubated for 3 min at RT, and centrifuged for 10 min at 11000 g at 4ºC. The supernatant was subjected to SEC analysis.

**Proteinase-K (PK) digestion**

Amyloid fibrils were generated in the presence or absence of 0.1% Brij-58 using the above described procedure, pelleted by centrifugation at 20,600 g for 30 min at 4°C, and washed twice with PBS [10 mM sodium phosphate (pH 7.4) and 150 mM NaCl]. Limited proteolysis of the amyloid fibrils and proteins monomers was performed using a previously published protocol with some modifications [7]. Briefly, PK from Tritirachium album was dissolved with distilled water at a protein concentration of 100–200 µM, just prior to use. The PK solution was diluted in distilled water to 1, 0.1, or 0.01 µM and mixed with a 9 volume of the protein solution that contains approximately 20-µM amyloid fibrils or protein monomers in PBS. The PK digestion reaction was allowed to proceed at 37°C for 1 hour and stopped by the addition of 5×sample buffer [0.25 M Tris-HCl (pH 6.8), 10% SDS, 0.5% Bromophenol Blue, 0.5 M DTT, and 50% glycerol] with boiling for 10 min. The PK-resistant fragments were analyzed by standard SDS-PAGE with Coomassie Blue Staining.

**GuHCl-unfolding experiment**

GuHCl-unfolding experiment of amyloid fibrils was performed using a previously published protocol with some modifications [8]. Initially, amyloid fibrils were generated without Brij-58 using the above described procedure, dispensed into aliquots, and pelleted by centrifugation at 20,600 g for 30 min at 4°C. Subsequently, amyloid fibrils were resuspended with various incubation buffers [20 mM HEPES-NaOH (pH 7.2), 1 mM EDTA, 0.01% NaN_3_, various concentrations of GuHCl (0–6 M), and 0, 0.1, or 2.5% Brij-58] at a protein concentration of 20 µM. The reaction mixture was incubated at 37°C for 24 hours, and diluted 4-fold with a ThT buffer [20 mM HEPES-NaOH (pH 7.2), 1 mM EDTA, 0.01% NaN_3_, 25 µM ThT, and various concentration of GuHCl (0–2.0 M)] to adjust the final GuHCl concentration to 1.5 M just prior to the fluorescence measurement. ThT fluorescence of the solutions was measured on the TECAN Infinite 200 F Nano+ plate reader as described above. The GuHCl-unfolding curves were analyzed using a linear polymerization model [9]:

$$F\left[ GuHCl \right]=s\times\sum_{i=1}^{\infty} i\left[ P_{i} \right]=s\times\left( \left[ M \right]_{0}-\left[ M \right] \right)=s\times\left( \left[ M \right]_{0}-\frac{2\left[ M \right]_{0}K_{a}+1-\sqrt{4\left[ M \right]_{0}K_{a}+1}}{2{\left[ M \right]_{0}K_{a}}^{2}} \right)$$

$$K_{a}=exp\left( \frac{{\Delta G}_{Am-U}-m\times[GuHCl]}{\mathrm{RT}} \right)$$

In the curve fitting, *∆G* (kcal/mol) was a locally varying parameter, while *m* (kcal/mol M) and *s* (a.u./µM) were globally fixed parameters. [M]_0_ was fixed to 20 µM. The best fit ∆G values were −8.13 ± 0.58 (0% Brij-58), −8.41 ± 0.61 (0.1% Brij-58), and −7.94 ± 0.55 (2.5% Brij-58). The best fit values for *m* and *s* were −0.80 ± 0.12 and 721 ± 38, respectively.


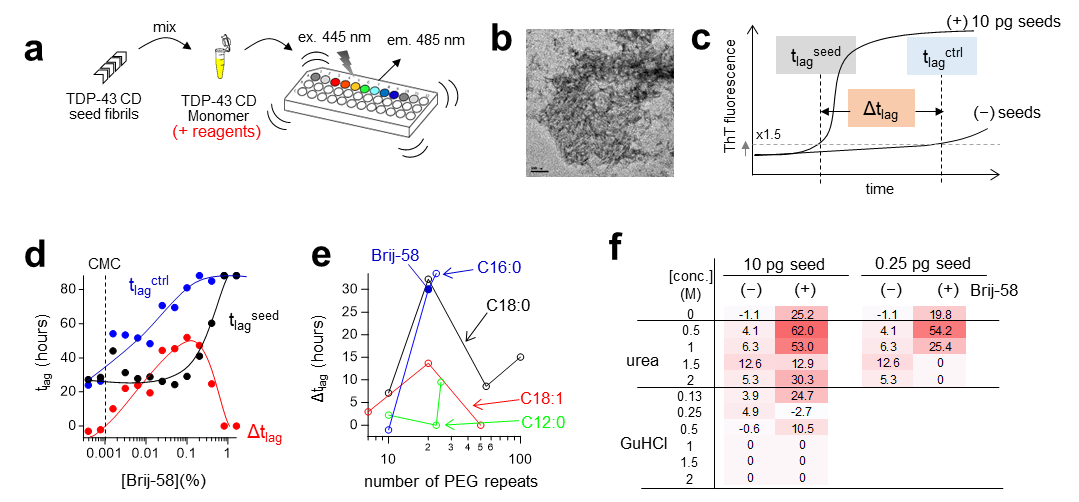


# **Figure S1 (related to Figures 1a, b)**: Development of an ultrasensitive TDP-43 SAA.

1. Schematic representation of SAA. Seed fibrils generated from a TDP-43 CD fragment were mixed with the same protein fragment in a monomeric conformation. Propagated protein aggregation by the seeds was monitored by the change in ThT fluorescence.
2. A representative transmission electron microscopy (TEM) image of seed fibrils is shown.
3. ∆t_lag_ is defined as the difference in the lag time of protein aggregation between the presence and absence of seeds (t_lag_^seed^ and t_lag_^ctrl^, respectively). The lag time is defined as the time when the ThT fluorescence reaches 150% of the initial value.
4. ∆t_lag_, t_lag_^seed^, and t_lag_^ctrl^ at various concentrations of Brij-58 are shown. Solid curves are drawn to guide the eye. **Supplemental note:** Brij-58 showed an optimal concentration range of 0.02%–0.2%. Above this range, ∆t_lag_ gradually decreased due to an extended t_lag_^seed^, suggesting that higher concentrations of Brij-58 inhibit seed-dependent aggregation. Conversely, below this range, ∆t_lag_ gradually decreased due to a shortened t_lag_^ctrl^, suggesting that lower concentrations of Brij-58 are insufficient to inhibit the self-aggregation of TDP-43 monomers. When the concentration dropped below 0.001%, ∆t_lag_ reached a value of 0. Interestingly, this value closely aligns with the CMC of Brij-58 [10], suggesting that the micelle formation plays a role in its MoA.
5. A screening result of Brij-58 analogs, which harbors varying repeats of PEG (5–100) and different structures of fatty acids (C12:0, C16:0, C18:0, and C18:1) at a concentration of 0.1%, is present. The conjugation of 20–23 PEG repeats with either palmitic acid (C16:0) or stearic acid (C18:0) yielded the best activity.
6. A list of the ∆t_lag_ values at different concentrations of urea (0.5–2.0 M) and GuHCl (0.13–2.0 M) with or without 0.1% Brij-58 is provided. **Supplemental note:** In efforts to further enhance the sensitivity of the TDP-43 SAA, we investigated the combined effect of Brij-58 and denaturants. This was inspired by a previous study that showed the addition of urea, but not guanidine hydrochloride (GuHCl), preferentially inhibits the self-aggregation of Aβ monomers over seed-dependent aggregation [11]. In line with the finding, adding 0.5–1.0 M urea to the TDP-43 SAA further increased the ∆t_lag_ value.

**
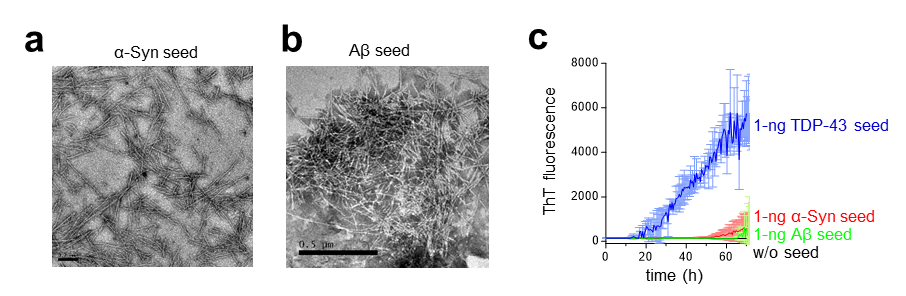
**

# **Figure S2 (related to Figure 1b):** Seed-specificity of the enhanced TDP-43 SAA.

(**a, b**) TEM images of α-synuclein and Amyloid-β seeds used in the experiment. (**c**) Time courses of TDP-43 protein aggregation propagated by 1 ng TDP-43, α-synuclein, or Amyloid-β seeds.


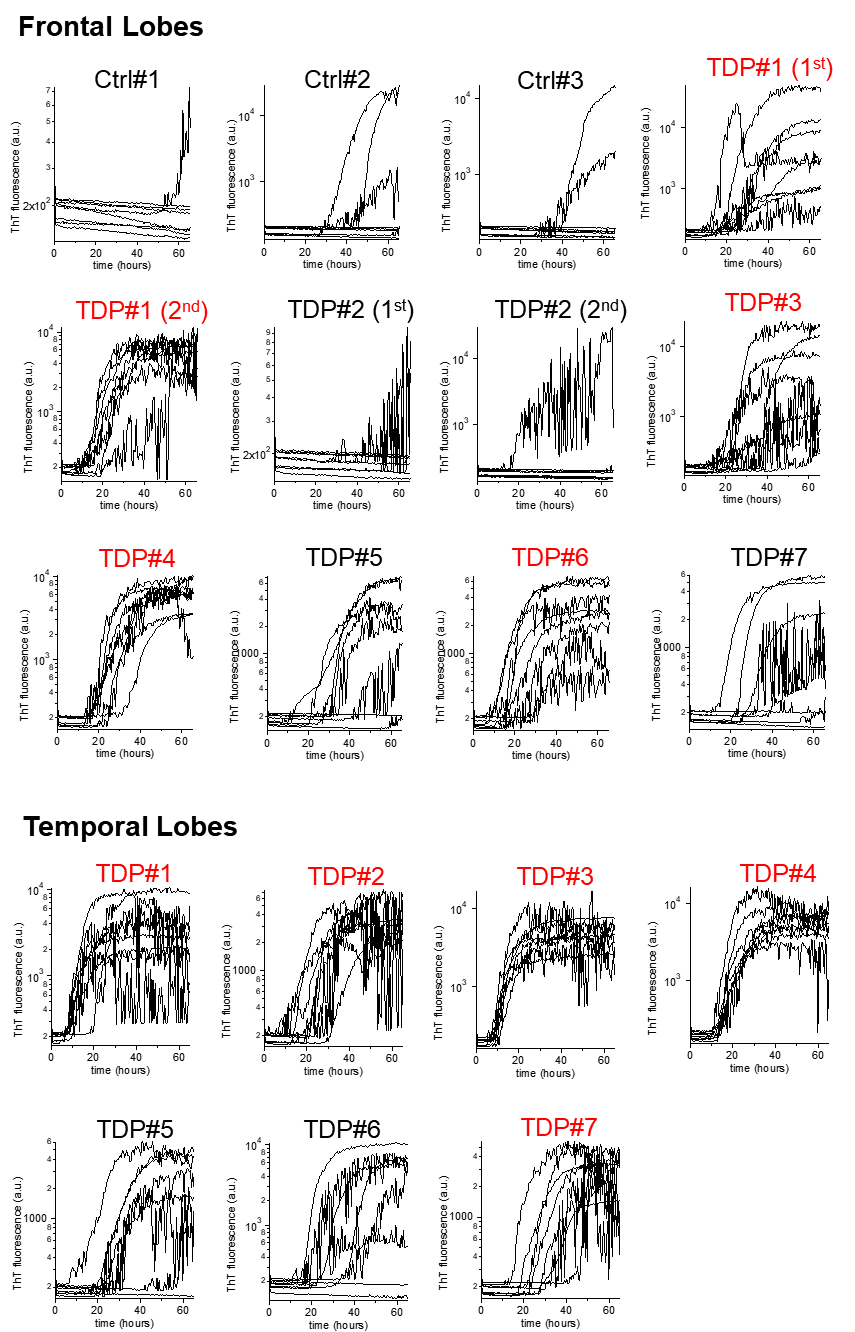


# **Figure S3 (related to Figure 1d)**: Original time courses of ThT fluorescence in the presence of patient-derived brain homogenates (1:250 dilution). Time courses of ThT fluorescence in the presence of patient-derived brain homogenates at a 1:250 dilution ratio (n = 8). Samples at P < 0.05 by Dunnett’s test versus control without brain homogenate are highlighted by red font.


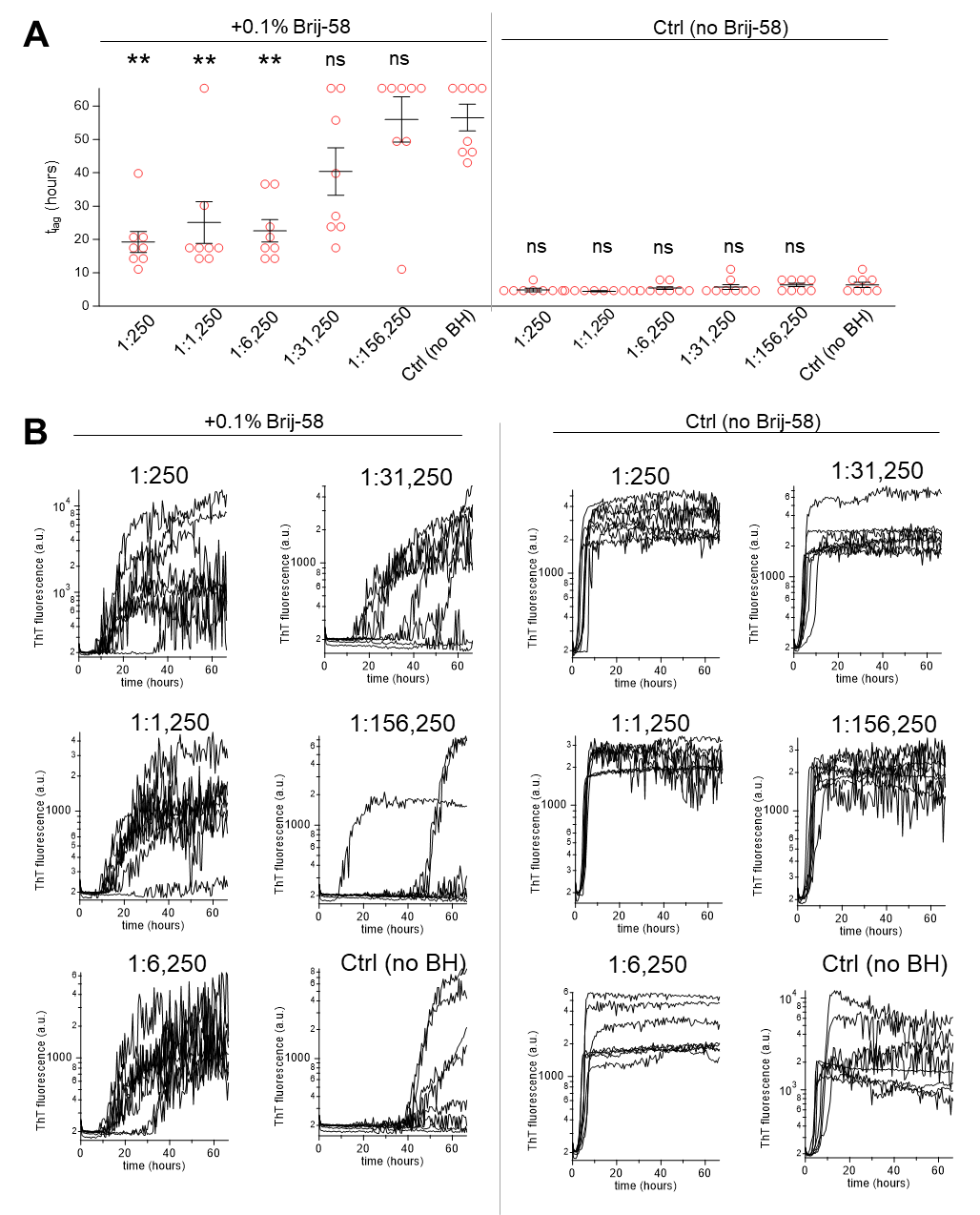


# **Figure S4 (related to Figure S3)**: Effects of Brij-58 in the detection of TDP-43 aggregates from patient-derived brain tissues.

(**a**) This panel shows the t_lag_ values of the TDP#1 patient’s lateral lobe-derived homogenate at various dilution ratios in the presence or absence of 0.1% Brij-58. The average ± S.E. from 8 measurements is provided (*P < 0.05, **P < 0.005, Dunnett’s test compared to the control without no brain homogenate). (**b**) This panel shows the original time courses of ThT fluorescence corresponding to Figure A.

**
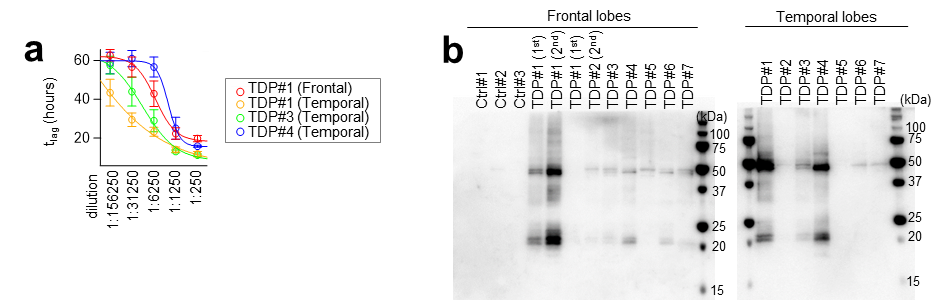
**

**Figure S5 (related to Figures 1D and 1E)**: Detection of TDP-43 aggregates in patient-derived brain tissues using SAA and immunoblot.

(**a**) This panel shows the t_lag_ values of patient-derived brain homogenates at various dilution ratios. The average ± S.E. from 8 measurements is provided. The original time courses of ThT fluorescence are provided in Figure S5.

(**b**) This panel displays immunoblot images of patient-derived brain homogenates probing phosphorylated TDP-43 at Ser409/410. Consistent with previous studies [12], immunoblot analysis detected two distinct bands at approximately 40–50 kDa and 20–25 kDa, corresponding to full-length and C-terminal fragments, respectively, in the sections from the FTLD-TDP patients, but not from the control patients. Notably, the intensities of these two bands correlated well with the seeding activity measured by SAA.


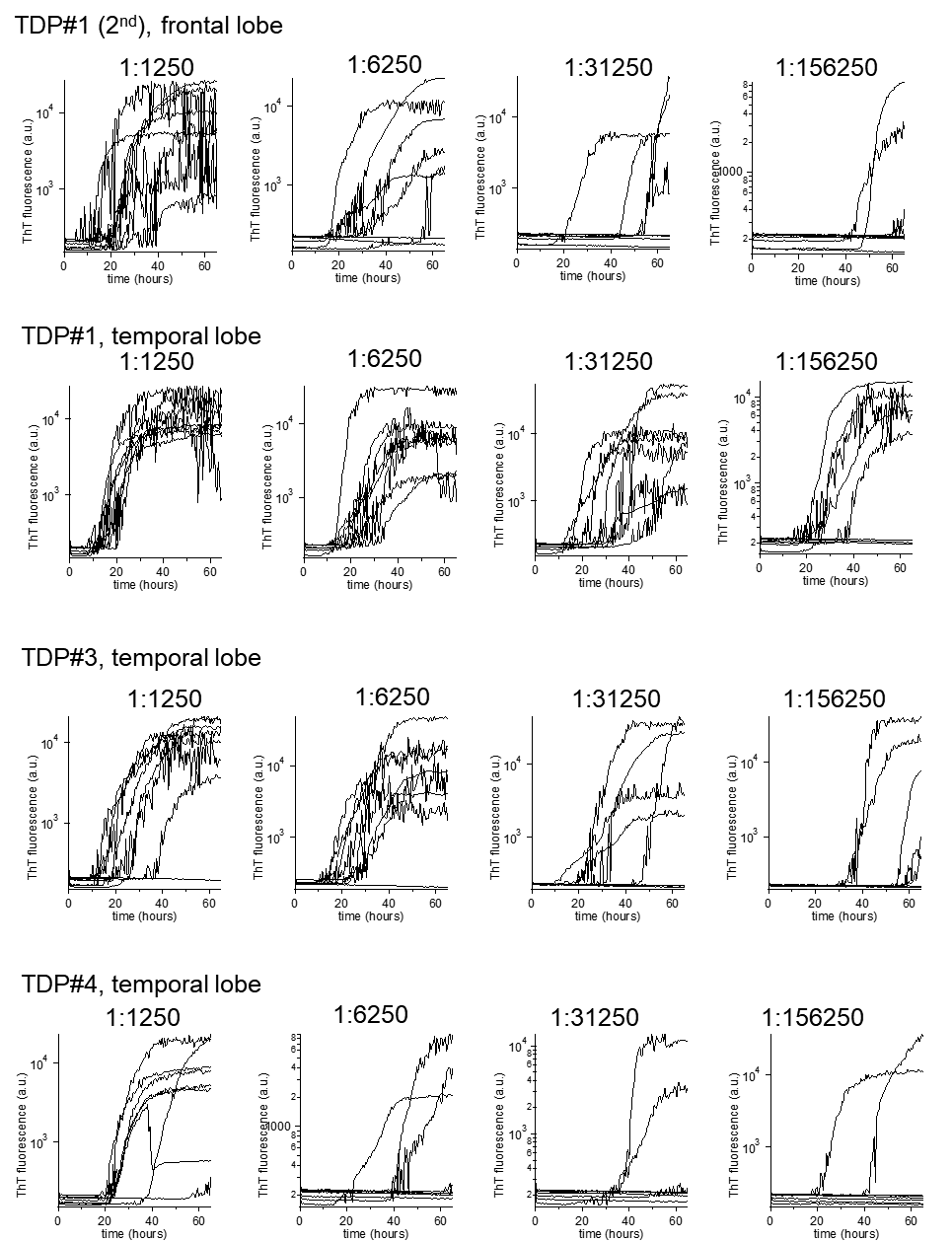


# **Figure S6 (related to Figure S5a)**: Original time courses of ThT fluorescence in the presence of patient-derived brain homogenates (1:1250–156250 dilutions)

# **
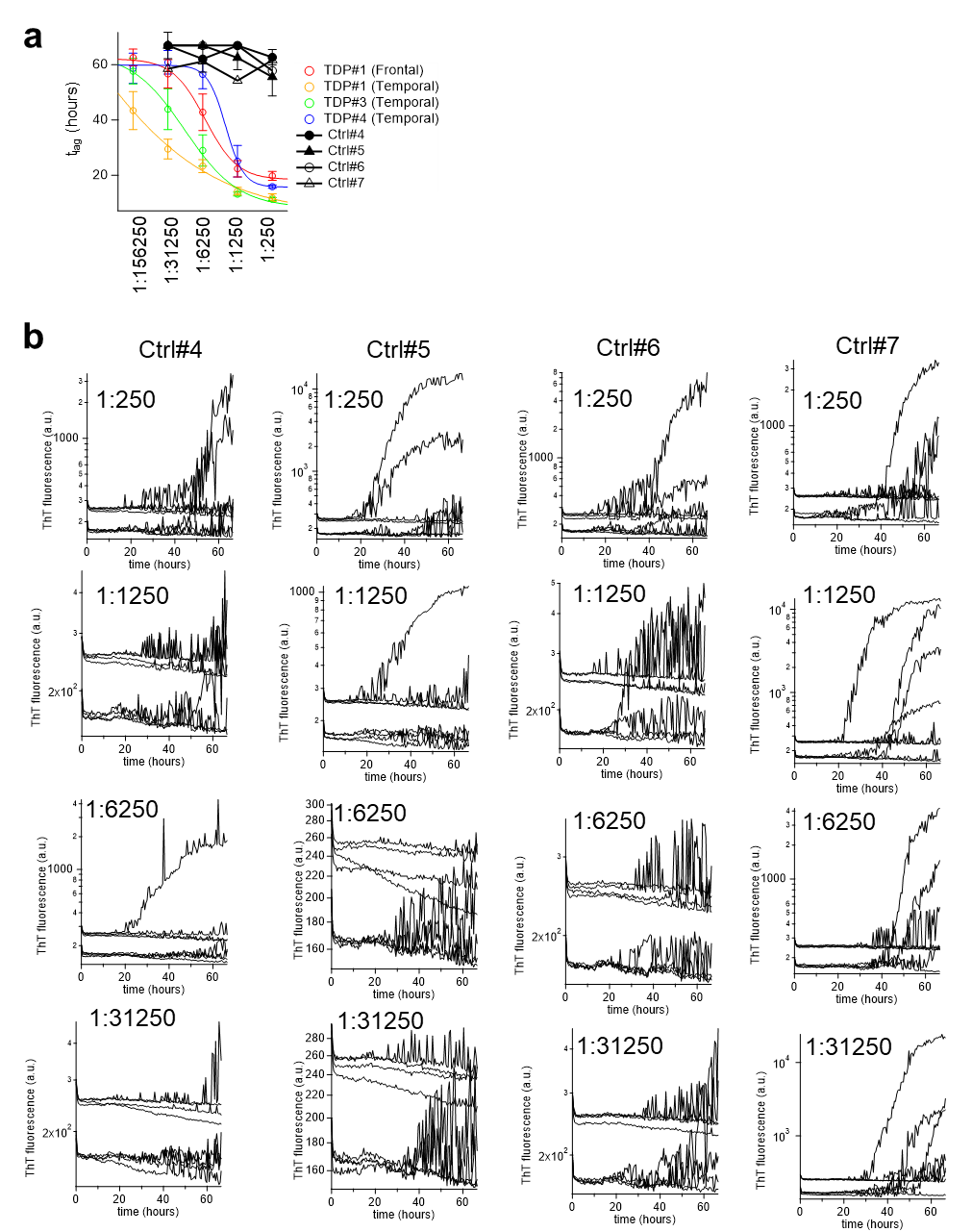
**

# **Figure S7 (related to Figures 1d and S5a):** A result of TDP-43 SAA for four neurologically healthy controls in a second cohort.

(**a**) This panel shows the t_lag_ values of the four neurologically healthy control patient’s frontal lobe-derived homogenate at various dilution ratios. The results were overlaid on Figure S5a. The average ± S.E. from 8 measurements is provided. (**b**) This panel shows the original time courses of ThT fluorescence corresponding to Figure A.


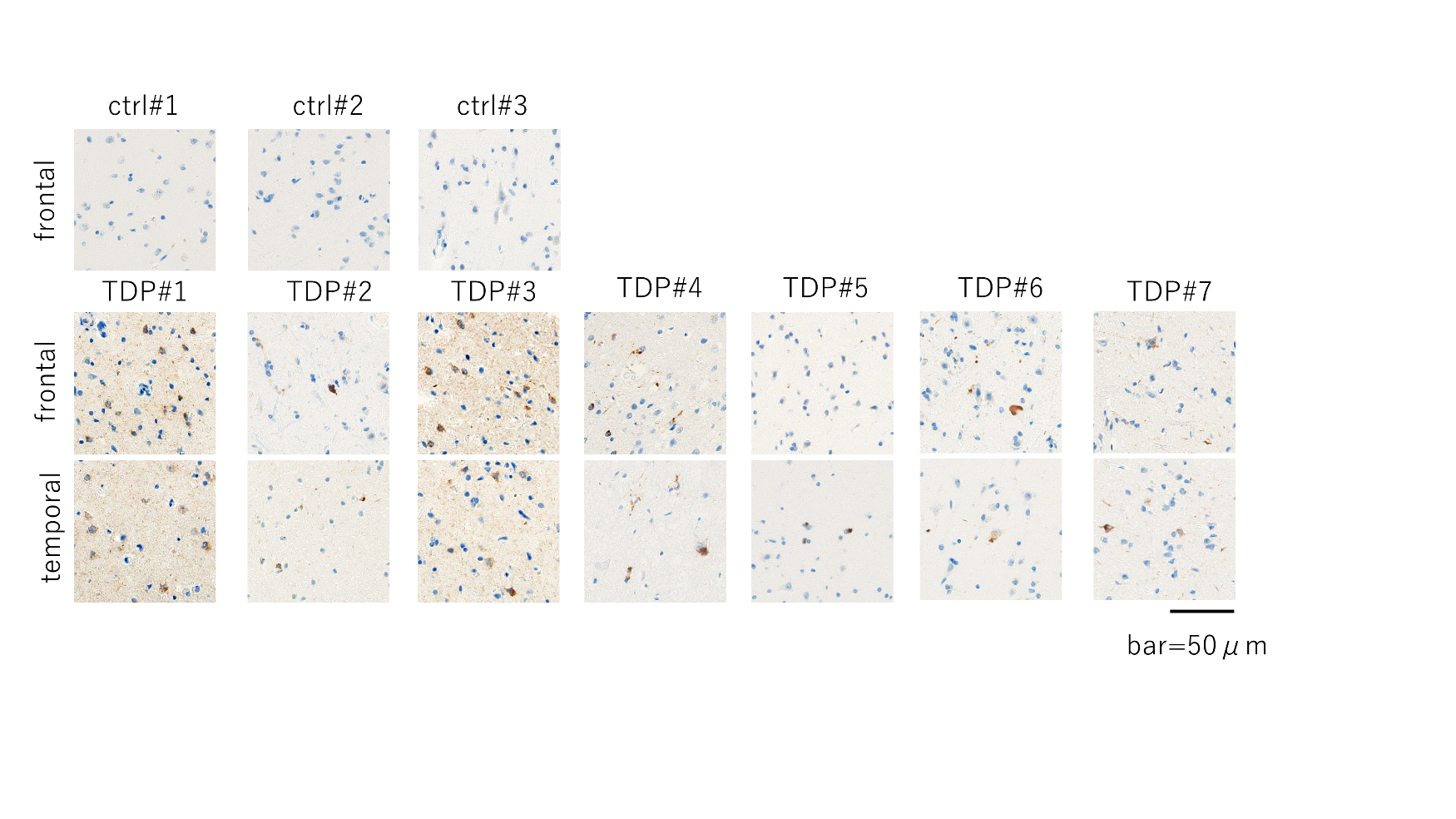


# **Figure S8 (related to Figure 1f):** IHC images of brain tissues probing phosphorylated TDP-43 at Ser409/410.


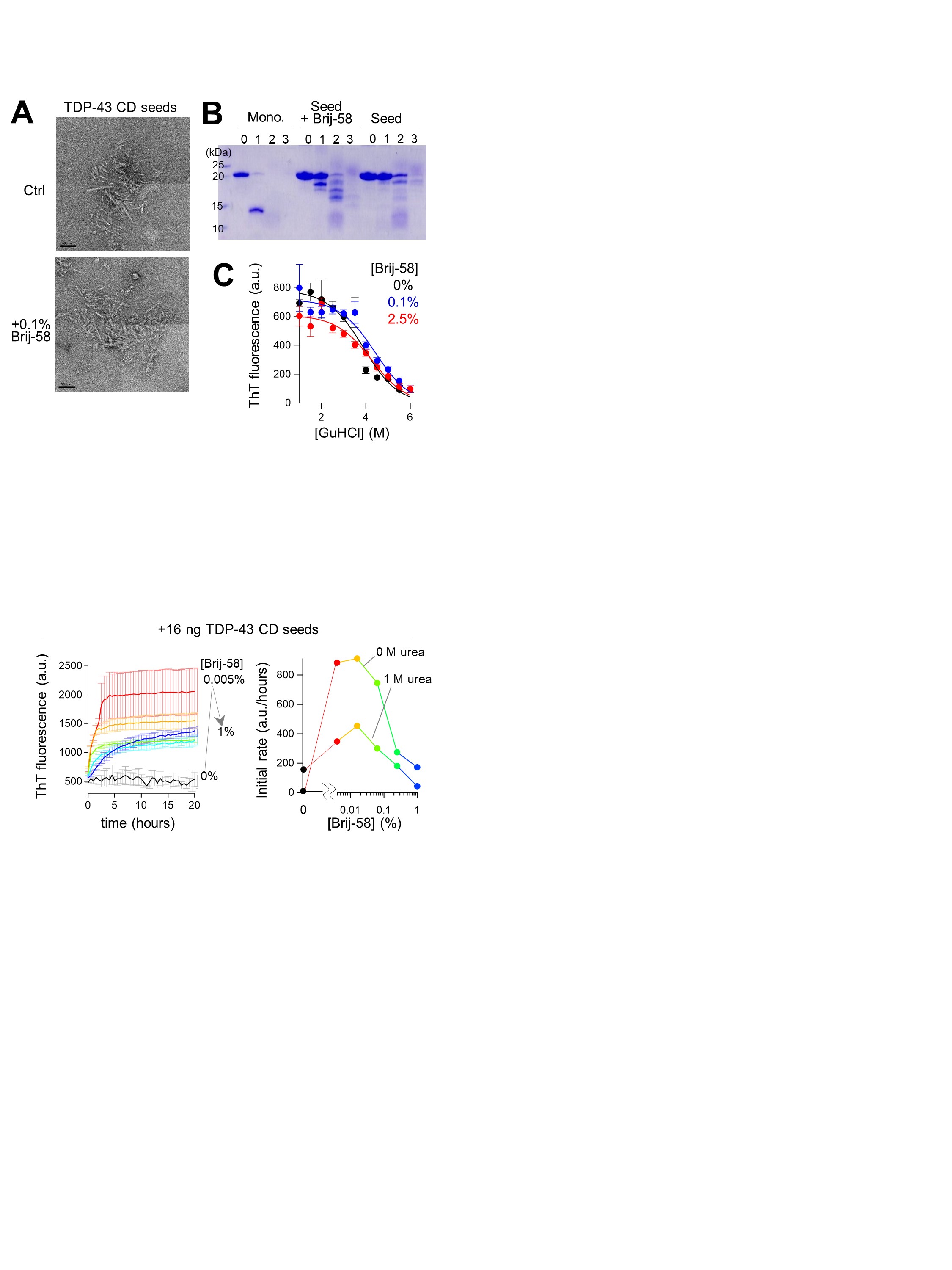


**Figure S9:** Brij-58 dramatically accelerates amplification of TDP-43 seed amplification. (left panel) time courses of ThT fluorescence in the presence of 16 ng seeds. (right panel) A plot of the initial rates of seed-dependent aggregation versus the Brij-58 concentrations derived from the left panel (without urea) or Figure S10 (with 1 M urea).

**Supplemental note:** To directly evaluate whether Brij-58 interferes with seed-dependent aggregation, we conducted an SAA using high concentrations of seeds (16 ng) under a quiescent condition [4]. In the absence of Brij-58 or urea, the rate of seed-dependent aggregation was nearly zero (right panel, 0 M urea), indicating that the TDP-43 monomers adopt a liquid droplet structure incapable of undergoing seed-dependent aggregation. Upon the addition of 0.005%–0.05% Brij-58, the reaction rate increased dramatically, exceeding the efficiency of 1 M urea (left panel, 0 M urea). Above 0.05%, the reaction rate began to decrease, consistent with the results shown in Figure 1F. Thus, Brij-58 at moderate concentrations accelerates seed-dependent aggregation, suggesting that the Brij-58/TDP-43 complex is capable of undergoing effective seed-dependent aggregation.


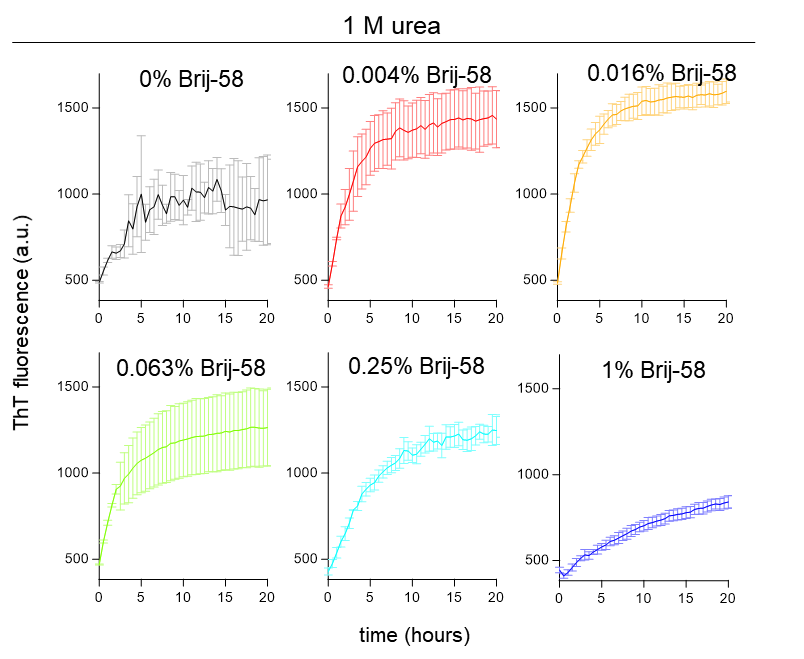


# **Figure S10 (related to Figure S9)**: Original time courses of ThT fluorescence in the presence of 16 ng seeds and 1 M urea

#
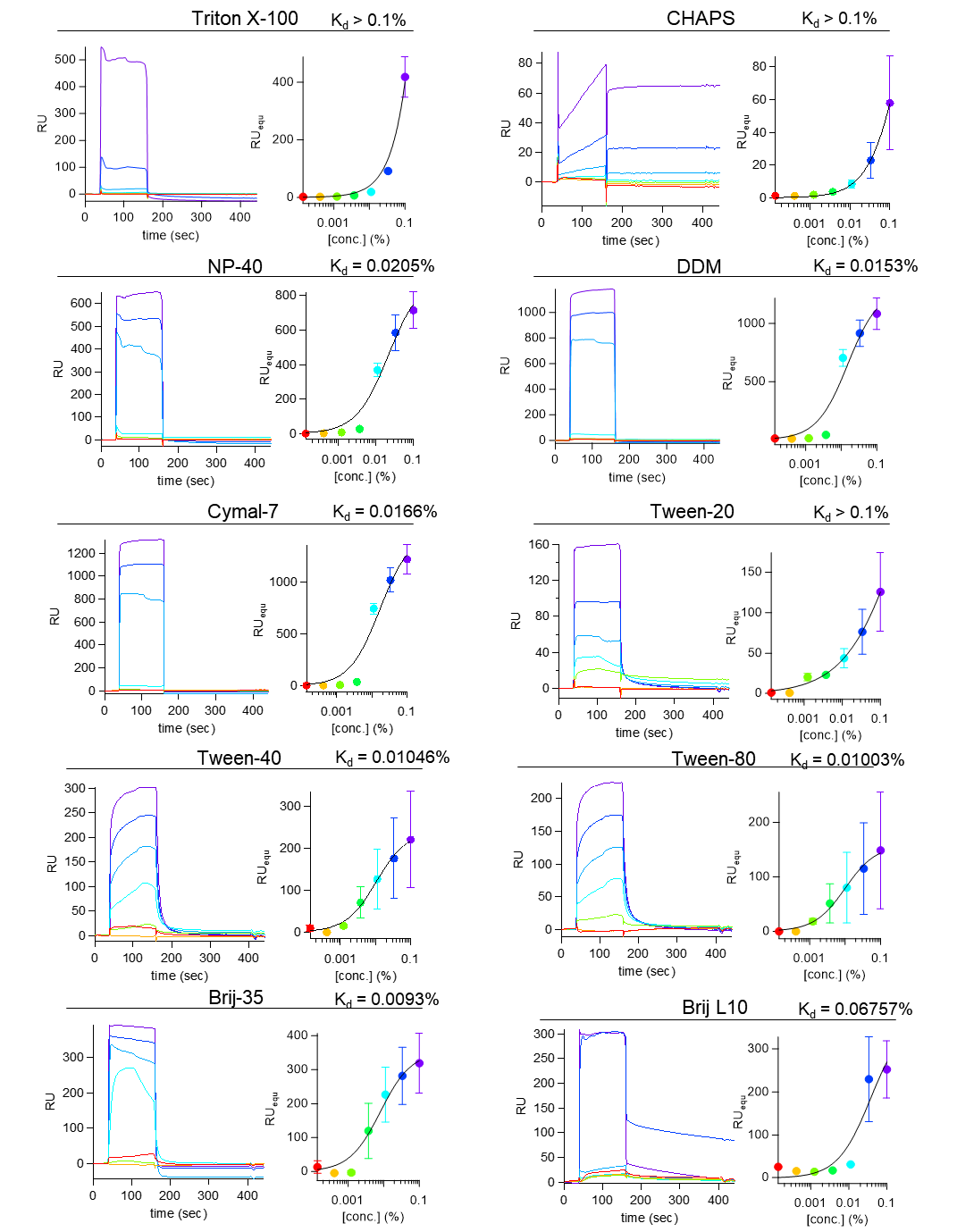


**Figure S11 (related to Figure 1g)**: SPR sensorgrams and dose-response curves of surfactant
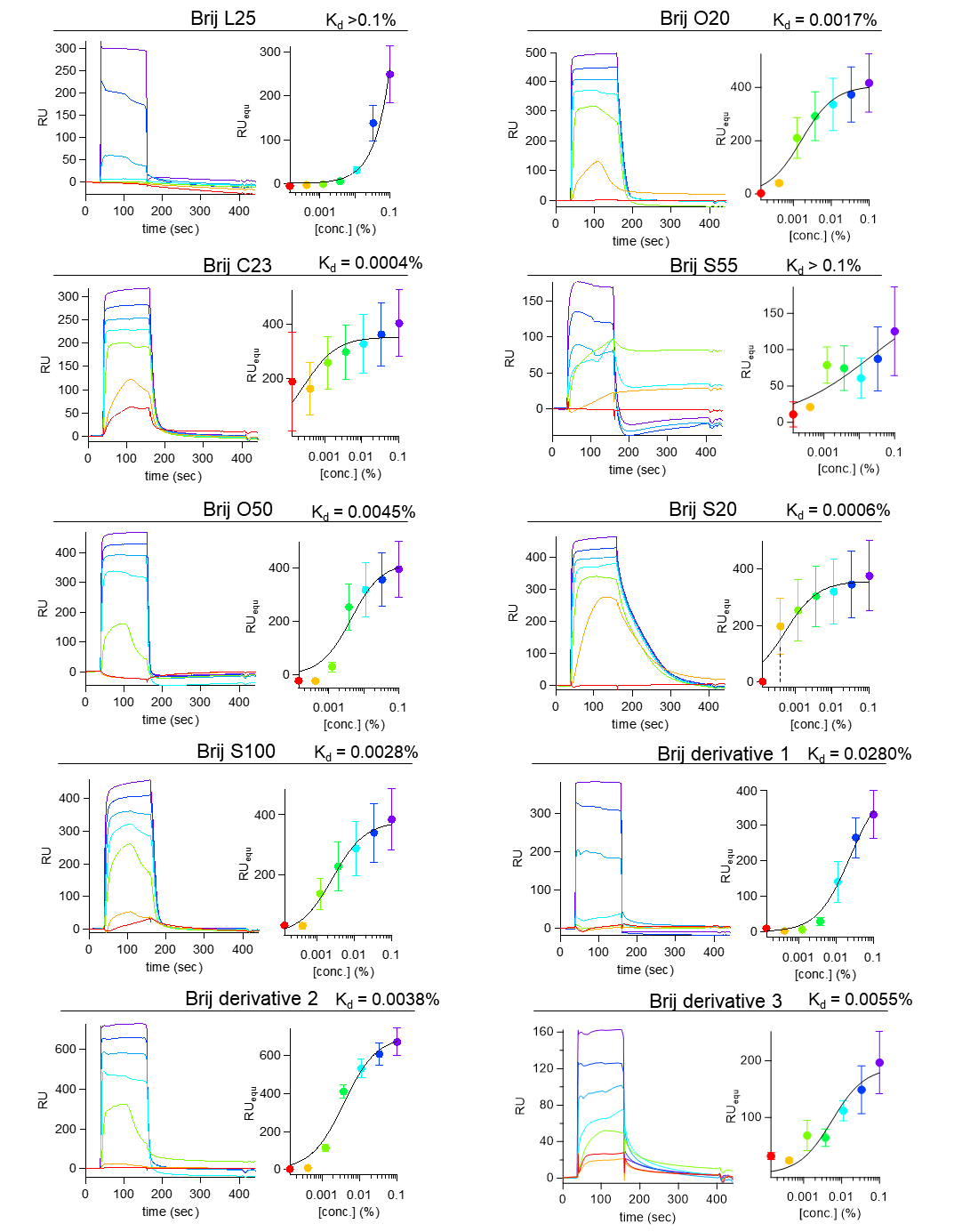
. The solid lines represent the least-square fits to sigmoidal functions.

# **
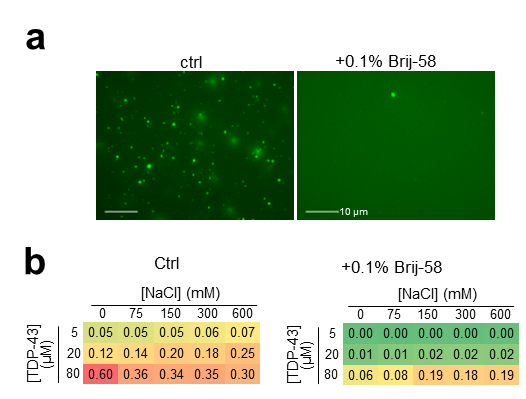
**

# **Figure S12**: Liquid droplet assay

(**a**) Fluorescence images of 20 µM fluorescein-labeled TDP-43 CD at 300 mM NaCl with or without 0.1% Brij-58 are present. (**b**) A list of turbidity at 600 nm of solutions containing various concentrations of TDP-43 CD and NaCl with or without 0.1% Brij-58 is present.

**Supplemental note**: It is known that TDP-43 monomers form liquid droplets under physiological buffer conditions [13,14]. We found that Brij-58 completely disrupted this liquid droplet structure (Figure S12A) and cleared the turbidity of the solution containing TDP-43 monomers (Figure S12B).


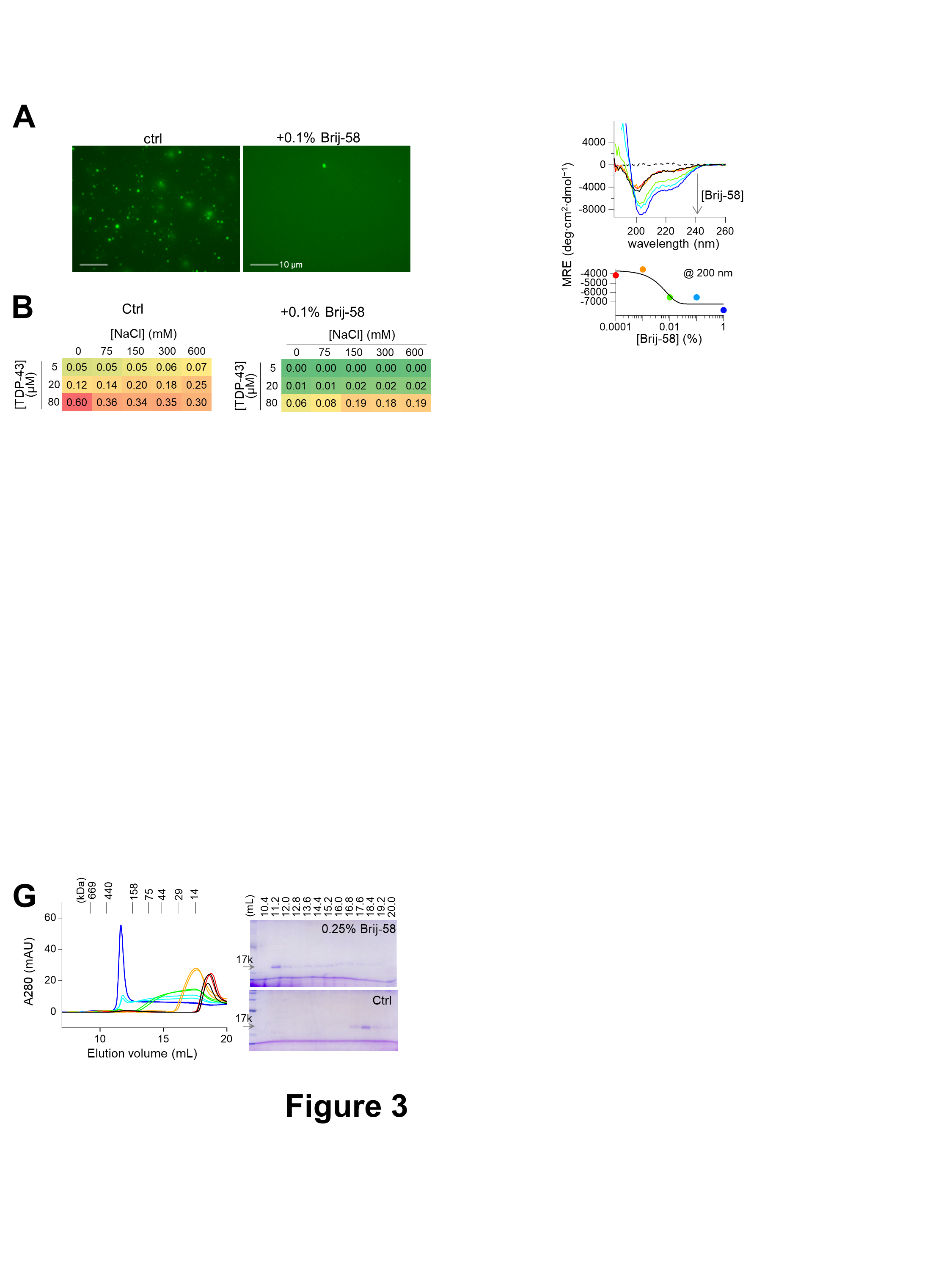


**Figure S13**: Far-UV circular dichroism (CD) analysis of the Brij-58/TDP-43 complex. (top panel) Far-UV CD spectra of TDP-43 CD with Brij-58 at various concentrations. (bottom panel) A plot of the CD values at 200 nm versus the Brij-58 concentration, derived from the top panel.

**Supplemental note**: Far-UV CD analysis showed that TDP-43 monomers undergo a conformational change from an intrinsically disordered structure into a partially ordered structure upon binding to Brij-58.

# **
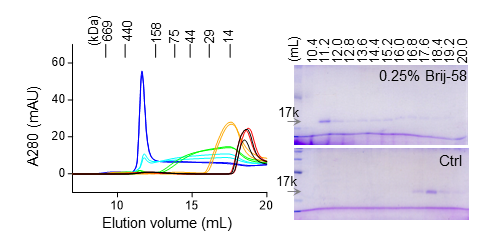
**

# **Figure S14 (related to Figure 1I)**: Size exclusion chromatography (SEC) analysis of the Brij-58/TDP-43 complex

(left panel, same as Figure 1i) SEC profiles of 20 µM TDP-43 CD mixed with 0% (black), 0.001% (red), 0.01% (orange), 0.05% (light green), 0.1% (light blue), and 0.25% Brij-58 (blue) for 3 min at 25ºC prior to analysis. (right panel) CBB-stained SDS-PAGE analysis of the SEC fractions.

**Supplemental note**: SEC analysis showed that the Brij-58/TDP-43 complex elutes as a single peak at 200 kDa, when 0.25% Brij-58 is mixed with the TDP-43 monomers before the analysis. Below 0.1%, the complex eluted as broad peaks with either fronting (0.1%) or tailing (0.05%), indicating that the Brij-58/TDP-43 complex dissociates during SEC, consistent with the rapidly reversible binding kinetics observed in the SPR analysis (Figure 1g). Therefore, TDP-43 and Brij-58 form a reversible 200 kDa micelle complex.


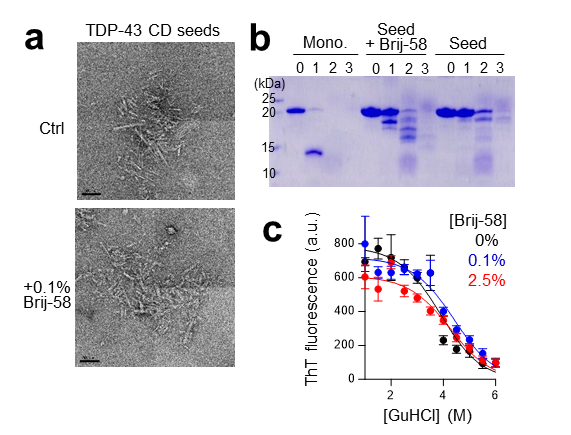


# **Figure S15**: Brij-58 does not significantly affect the structure or stability of seed fibrils.

(**a**) Representative TEM images of negatively stained TDP-43 seed fibrils generated with or without 0.1% Brij-58. Brij-58 did not alter the morphology of seed fibril.

(**b**) CBB-stained SDS-PAGE analysis of Proteinase K (PK)-resistant fragments of TDP-43 monomer (left) or seeds generated with (middle) or without 0.1% Brij-58 (right lanes). The proteins were treated with 0 (lane 1), 0.001 (lane 2), 0.01 (lane 3), or 0.1 µM PK (lane 4) at 37ºC for 1 h prior to SDS-PAGE analysis. Brij-58 slightly increases the susceptibility of seed fibrils to K digestion, suggesting that the fibril core is exposed to the solvent and thus is more accessible for binding by TDP-43 monomers.

(**c**) GuHCl-denaturation curves for TDP-43 seeds treated with 0 (black), 0.1 (blue), and 2.5% Brij-58 (red) at 37ºC for 24 h. The solid lines represent the least-square fits to theoretical denaturation curves. The best fit ∆G values were −8.13 ± 0.58 (black), −8.41 ± 0.61 (blue), and −7.94 ± 0.55 kca/mol (red). Brij-58 does not affect the overall stability of seed fibrils. Overall, Brij-58 does not significantly alter the structure or stability of seeds, contrasting with its substantial effects on TDP-43 monomers.


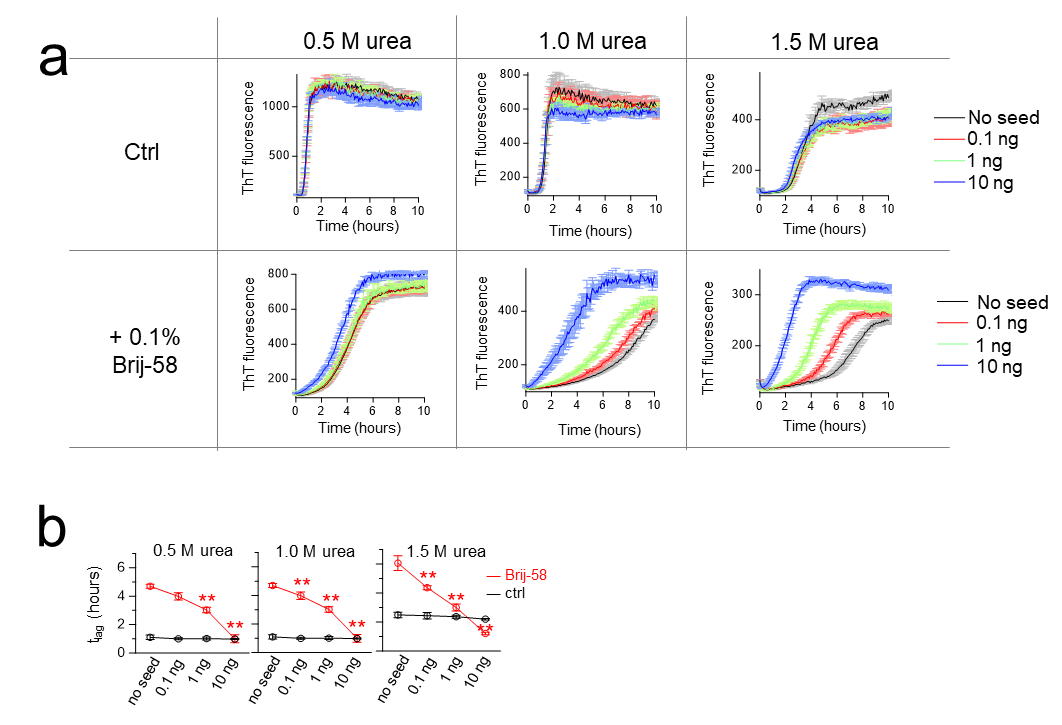


# **Figure S16 (related to Figure 1L)**: Brij-58 improves the detection limit of Aβ SAA.

(**a**) Time courses of ThT fluorescence at different concentrations of Aβ seeds. We developed an Aβ SAA using a method similar to one used for the TDP-43 SAA, employing 0.5, 1.0, or 1.5 M urea with or without 0.1% Brij-58. (**b**) the t_lag_ values derived from Figure A (**P* < 0.05, ***P* < 0.005, unpaired two-tailed t-test compared to without seed).

#
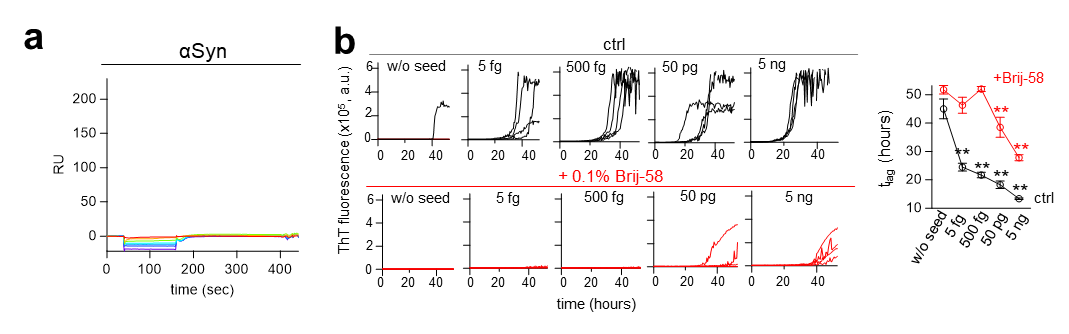
 **Figure S17**: Brij-58 did not bind to αSyn, nor improves the detection limit of αSyn SAA.

(**a**) SPR sensorgrams of Brij-58 binding to αSyn monomers. (**b**) (left panel) Time courses of ThT fluorescence of αSyn SAA at different seed concentrations, both without (black lines) and with 0.1% Brij-58 (red lines). (right panel) t_lag_ values derived from the left panel (**P* < 0.05, ***P* < 0.005, unpaired two-tailed t-test compared to without seed). Brij-58 reduced the detection limit of αSyn SAA from < 5 fg to 50 pg.

# **Supplemental References**

[1] A.M. Peterson, Z. Tan, E.M. Kimbrough, J.M. Heemstra, 3, 3′-Dioctadecyloxacarbocyanine perchlorate (DiO) as a fluorogenic probe for measurement of critical micelle concentration, Analytical Methods 7 (2015) 6877-6882.

[2] M. Masuda, N. Dohmae, T. Nonaka, T. Oikawa, S. Hisanaga, M. Goedert, M. Hasegawa, Cysteine misincorporation in bacterially expressed human alpha-synuclein, FEBS letters 580 (2006) 1775-1779. 10.1016/j.febslet.2006.02.032.

[3] M. Shirasaka, K. Kuwata, R. Honda, α-Synuclein chaperone suppresses nucleation and amyloidogenesis of prion protein, Biochemical and biophysical research communications 521 (2020) 259-264.

[4] R.P. Honda, K. Kuwata, The native state of prion protein (PrP) directly inhibits formation of PrP-amyloid fibrils in vitro, Scientific Reports 7 (2017) 562. 10.1038/s41598-017-00710-x.

[5] T.K. Nomura, K. Heishima, N. Sugito, R. Sugawara, H. Ueda, A. Yukihiro, R. Honda, Specific inhibition of oncogenic RAS using cell-permeable RAS-binding domains, Cell Chemical Biology 28 (2021) 1581-1589. e1586.

[6] W.M. Babinchak, R. Haider, B.K. Dumm, P. Sarkar, K. Surewicz, J.-K. Choi, W.K. Surewicz, The role of liquid–liquid phase separation in aggregation of the TDP-43 low-complexity domain, Journal of Biological Chemistry 294 (2019) 6306-6317.

[7] R. Honda, K. Kuwata, Evidence for a central role of PrP helix 2 in the nucleation of amyloid fibrils, The FASEB Journal 32 (2018) 3641-3652.

[8] R. Honda, Role of the Disulfide Bond in Prion Protein Amyloid Formation: A Thermodynamic and Kinetic Analysis, Biophys J 114 (2018) 885-892. 10.1016/j.bpj.2017.12.031.

[9] T. Narimoto, K. Sakurai, A. Okamoto, E. Chatani, M. Hoshino, K. Hasegawa, H. Naiki, Y. Goto, Conformational stability of amyloid fibrils of β 2-microglobulin probed by guanidine-hydrochloride-induced unfolding, FEBS letters 576 (2004) 313-319.

[10] A. Helenius, D.R. McCaslin, E. Fries, C. Tanford, [63] Properties of detergents, Methods in enzymology 56 (1979) 734-749.

[11] T. Weiffert, G. Meisl, S. Curk, R. Cukalevski, A. Šarić, T.P. Knowles, S. Linse, Influence of denaturants on amyloid β42 aggregation kinetics, Front Neurosci 16 (2022) 943355.

[12] M. Hasegawa, T. Arai, T. Nonaka, F. Kametani, M. Yoshida, Y. Hashizume, T.G. Beach, E. Buratti, F. Baralle, M. Morita, I. Nakano, T. Oda, K. Tsuchiya, H. Akiyama, Phosphorylated TDP-43 in frontotemporal lobar degeneration and amyotrophic lateral sclerosis, Annals of neurology 64 (2008) 60-70. 10.1002/ana.21425.

[13] A.E. Conicella, G.H. Zerze, J. Mittal, N.L. Fawzi, ALS mutations disrupt phase separation mediated by α-helical structure in the TDP-43 low-complexity C-terminal domain, Structure 24 (2016) 1537-1549.

[14] A. Molliex, J. Temirov, J. Lee, M. Coughlin, A.P. Kanagaraj, H.J. Kim, T. Mittag, J.P. Taylor, Phase separation by low complexity domains promotes stress granule assembly and drives pathological fibrillization, Cell 163 (2015) 123-133.
